# THERAPEUTIC DELIVERY OF ANG(1-7) VIA GENETICALLY MODIFIED PROBIOTIC: A DOSING STUDY

**DOI:** 10.1093/geroni/igz038.3071

**Published:** 2019-11-08

**Authors:** Christy S Carter

**Affiliations:** University of Alabama at Birmingham, Birmingham, Alabama, United States

## Abstract

Aging is associated with loss of diversity in gut microbiota leading to dysbiosis; a condition linked with cognitive/physical frailty. Age-related health benefits have been ascribed to the renin-angiotensin system (RAS), mediated via the angiotensin-converting-enzyme-2 (ACE2)/angiotensin (1-7) or Ang(1-7) axis. A genetically modified probiotic secreting Ang(1-7) or GMP-A, targeting the gut, has been described for diabetes and hypertension; however, we are the first to provide proof of concept for its use in aging. We report that GMP-A increases circulating levels of Ang(1-7) when orally-administered over 4 weeks in aged F344/BN rats. Ongoing experiments examine GMP-A impact on distal tissues (muscle/brain). GMP-A provides translational advantages including ease of administration, production cost, and burden for regulatory approval.

